# How to select a chiropractor for the management of athletic conditions

**DOI:** 10.1186/1746-1340-17-3

**Published:** 2009-03-10

**Authors:** Wayne Hoskins, Henry Pollard, Peter Garbutt

**Affiliations:** 1 Macquarie Injury Management Group, Department of Health & Chiropractic, Macquarie University, Sydney, NSW 2109, Australia

## Abstract

**Background:**

Chiropractors are an integral part of the management of musculoskeletal injuries. A considerable communication gap between the chiropractic and medical professions exists. Subsequently referring allopathic practitioners lack confidence in picking a chiropractic practitioner with appropriate management strategies to adequately resolve sporting injuries. Subsequently, the question is often raised: "how do you find a good chiropractor?".

**Discussion:**

Best practice guidelines are increasingly suggesting that musculoskeletal injuries should be managed with multimodal active and passive care strategies. Broadly speaking chiropractors may be subdivided into "modern multimodal" or "classical" (unimodal) in nature. The modern multimodal practitioner is better suited to managing sporting injuries by incorporating passive and active care management strategies to address three important phases of care in the continuum of injury from the acute inflammation/pain phase to the chronic/rehabilitation phase to the injury prevention phase. In contrast, the unimodal, manipulation only and typically spine only approach of the classical practitioner seems less suited to the challenges of the injured athlete. Identifying what part of the philosophical management spectrum a chiropractor falls is important as it is clearly not easily evident in most published material such as Yellow Pages advertisements.

**Summary:**

Identifying a chiropractic practitioner who uses multimodal treatment of adequate duration, who incorporates active and passive components of therapy including exercise prescription whilst using medical terminology and diagnosis without mandatory x-rays or predetermined treatment schedules or prepaid contracts of care will likely result in selection of a chiropractor with the approach and philosophy suited to appropriately managing athletic conditions. Sporting organizations and associations should consider using similar criteria as a minimum standard to allow participation in health care team selections.

## Background

Chiropractic has travelled a difficult path to recognition in recent years. This is particularly true in the sports realm. The authors of this commentary have a combined experience of sports representation at the national and international level as practitioners, writing and teaching academic sports chiropractic programs and published research in sports chiropractic. The collective experience of this group has led to the formulation of various operational guidelines that may be useful to other healthcare practitioners. However, it is important to recognise that the views expressed in this work are those of the authors and not any body to which they are affiliated.

This paper was inspired by interaction in a recent tutorial provided by sports chiropractors to Australasian College of Sports Physician (ACSP) Registrars as part of the official ACSP training program. During the exchange it became clear that there was a considerable communication gap between the professions and the question was raised: "how do you find a good chiropractor?" As with all professions there is a difference in practitioner skill and expertise, although this difference is perhaps more diverse in chiropractic [1,2]. For many of the registrars this was their first interface with a chiropractor, largely due to medical schools and hospitals within Australia being separate from chiropractic university education and training. Much of what the registrars knew was from second hand information although it was apparent that the registrars understood that there were "good and bad chiropractors".

Chiropractors and manual therapists are an integral part of the treatment of musculoskeletal injuries and disabilities. Referring practitioners should have some understanding of the various types of chiropractors and practitioners and the various treatments and modalities used. The aim of this commentary is to provide a quick reference guide for non-chiropractors to use in the selection of or referral to a chiropractor for the management of athletic conditions.

## Discussion

Chiropractic is a very broad primary contact healthcare profession with an assortment of different technique and philosophical groups [3]. A distinction has been made such there are those who are called "modern multimodal" (MMM) chiropractors and those who are "classical" chiropractors [4,5]. Hoskins et al. [4] have said that MMM chiropractic management incorporates components of passive and active care to address both the acute inflammation/pain phase and the chronic/rehabilitation/injury prevention phase. Management often begins with acute injuries and continues through to the sub-acute or chronic phase (if necessary), with management changing along the path of patient recovery. After the pain and function has normalised, many chiropractors and their patients choose to pursue preventative strategies sometimes referred to as wellness or performance based treatment. Not all practitioners pursue this approach. Such MMM management typically incorporates a combined approach of various manual therapy procedures with an emphasis on high-velocity low-amplitude manipulation, soft tissue and stretching techniques, rehabilitation and therapeutic exercises, and non-local biomechanical improvement to improve kinematic and kinetic chain function. Other modalities used include taping, physical therapies, electrotherapeutics, acupuncture, gait retraining, nutrition, footwear/ergonomic/training advice and exercise/cross training programs. With spine-based management, psychosocial and other risk factors for chronicity are typically assessed at 4–6 weeks to avoid progression to chronic debilitation, and similar approaches are important in the prevention of chronic extremity injury. It should be noted that the MMM approach is characteristic of the preferred sports chiropractor [1] and is provided to all candidates in undergraduate university education and training [6,7]. The MMM approach should be evidence based where data is available to inform management.

Alternatively, classical chiropractors do not generally provide a multimodal treatment strategy incorporating soft tissue approaches, physical therapies, active rehabilitation, therapeutic exercise or pharmacological recommendation [1]. These practitioners follow historically derived approaches to patient management being typically unimodal in nature [1,4]. They characteristically utilise a manipulation-only approach and often a spine-only approach to address the osseous/joint component of patient complaints and the reflex neurophysiological changes secondary to the osseous/joint change [8,9]. However, athletes typically need direct active and/or passive management strategies to all tissues: osseous, muscle, ligament and fascia at a spine and extremity level in a patient centred approach to care rather than a practitioner centred approach.

Subsequently, many of the classical practitioners that subscribe to certain technique monotherapies and dated philosophical beliefs are not suited to the management of athletic injuries or athletic populations, whilst we contend that many MMM chiropractors are so suited [1,9]. This is largely a result of classical chiropractors choosing not to implement a management strategy that addresses pain and acute inflammation in conventional ways, along with rehabilitation or exercise prescription [1], all of which are fundamental for the management of most sporting injuries. The classical chiropractor often differentiates themselves from MMM chiropractors on the basis that they follow the "philosophical" view, or more correctly the historical view of chiropractic. It should be noted that philosophy is a desire to gain knowledge, to search and has nothing to do with the "belief" system that many chiropractor's refer to as their "philosophy". This belief system should also be supported by evidence too. It should also be noted the classical style of chiropractic, as with any older form of approach in health care is being utilised by fewer chiropractors every year.

Identifying what part of the philosophical management spectrum a chiropractor falls in is not clearly evident or publicized. One cannot simply look in the "Yellow Pages" to determine these facts. As a result, random selection or referral to a chiropractor may be met with disappointing results. Table 1 describes the key criteria and principles that we feel are important in the identification of an appropriate chiropractor. This table describes the attributes and management strategies that are desirable if they are to be suitable for the management of athletic injuries. These attributes can be determined quickly during a short phone inquiry by referring doctor or patient.

**Table 1:** Key criteria and principles which the chiropractor should demonstrate if they are to be suitable for the management of athletic injuries

Minimum treatment time 15–20 minutes
Treatment is multimodal in nature

Treatment should contain active (exercises) and passive components

No mandatory x-rays required for treatment

No predetermined treatment schedules or prepaid contracts of care

Use of medical terminology and diagnosis

* The criteria and principles are based on direct questions provided by Australasian College of Sports Physician Registrars

Although treatment should be individualized, the time of treatment should be no less than 15–20 minutes, which coincidentally is the requirement for the Australian Medicare allied health referral system [10]. Although the type of treatment rendered is often a function of time, treatment should also be multimodal in nature. The manual therapy and chiropractic literature and education suggests the multimodal approach as the logical way to patient management. Clearly this form of management cannot be rendered within a 2–5 minute treatment time frame that some high volume practitioners operate under, particularly those chiropractors that are spine-only and manipulation-only in their approach. Neither is it conceivable that risk assessment nor re-evaluation for rational continuation of treatment can be done in this space of time. In the absence of evidence that suggests that 2–5 minute treatment is better than multimodal treatment of 15–20 minute duration, the multimodal approach should be followed. It is up to the 2–5 minute practitioners to provide evidence that their particular approach is superior to the other longer treatment, which is supported by a larger body of published literature. Management of spine and extremity conditions should be with local and often non-local management strategies. Such approaches are consistent with approaches by other healthcare professions such as physiotherapy and osteopathy, although differences exist in application [11].

The chiropractor like all practitioners should operate utilising an evidence based approach for all components of therapy including the provision of radiographs [12-14]. Essentially the patient should not receive mandatory x-rays as a requirement of treatment unless indicated [13]. They should also have knowledge of when to refer for advanced forms of imaging such as CT, MRI and diagnostic ultrasound and know the indications of when to refer [12]. Chiropractors should also be conversant with and be expected to communicate with patients and referring practitioners through standard medical terminology [15]. Diagnosis and explanation should be provided and expressed in terms of these medical descriptions, not 'unique' chiropractic language, descriptions, or jargon [15]. This will assist with corresponding with all members of the health care team with language and descriptions that everyone understands. An inability or unwillingness to do so demonstrates and incapability to work in a team based, multidisciplinary environment, a long-standing criticism of some chiropractors [1].

Furthermore, the chiropractor should not provide predetermined treatment schedules or prepaid contracts of care, which do not fit with individualized and patient centred approaches to management [16]. Management should be based on a case-by-case basis. The 'one size fits all' pre-paid contracts and other practice management schemes are strongly discouraged by the leaders and majority of the chiropractic profession and have been the source of many complaints in Australia [17].

All chiropractors are familiar with risk management for safety and medicolegal reasons and should implement them actively in the provision of care [18,19]. This will result in appropriate practices for patient screening and selection for treatment and choice of treatment modality. The chiropractor should perform a complete and thorough history and physical examination prior to deciding to embark upon treatment of the injured athlete, just like any other practitioner. Not limited to this, screening should include standardized orthopaedic, neurological, joint based assessment (e.g. static and motion palpation) and other testing procedures, assessment of vital signs and – despite recent literature – vertebro-basilar insufficiency testing where appropriate for medicolegal purposes [20,21]. It is expected that athletes receive an appropriate individualized history in a traditional medical sense, which should assist with ruling out red flag conditions prior to the physical examination and other testing procedures. Additionally, it is likely that the sports specific history and examination should include analysis of training and competition needs, including specific biomechanical analysis/investigation of function commensurate with the level of play. Importantly, all practitioners should be aware of their limitations with certain conditions and partner with other medical team members to provide a full range of service to the athlete [4]. Only when such complimentary services are offered do we as practitioners truly provide patient centred health care.

## Summary

When trying to select or find a chiropractor to refer to, one would ask the questions of the chiropractor based on the characteristics listed in Table 1 to ascertain whether the chiropractor fits these criteria. It is the authors' opinion that a chiropractor possessing all of the criteria would be equipped with the approach and philosophy to appropriately manage athletic conditions. The authors also recommend that sporting organizations and associations use similar criteria as a minimum standard to allow participation in health care team selections.

## Conclusion

The purpose of this paper was to facilitate communication between the chiropractic and medical and allied health care professions in the attempt to maximise athlete patient care outcomes. When referring practitioners or athletic patients, following the quick and simple approach for assessment of a chiropractor's management approaches and philosophies, will likely find suitable practitioners committed to working together in a multidisciplinary approach to enhance the health of their athletic patients.

## Competing interests

The authors have no conflict of interest that is directly relevant to the content of this manuscript. No source of funding was used in the preparation of this manuscript.

## Authors' contributions

WH and HP presented the tutorial to the Australasian College of Sports Physician (ACSP) Registrars and conceived the idea of the paper. At a series of meetings, email and phone conversations WH, HP and PG further developed the discussion of the paper. All authors contributed to writing an initial draft document that reflected the collective thoughts and experiences of the participants. Over a course of further meetings, email and phone conversations, all authors contributed to the writing and re-writing of this paper. All authors made original contributions to the content of the final manuscript. All of the authors participated in the editing and revisions of the multiple drafts that existed between the initial and final draft. All authors read and approved the final manuscript.

## 
